# Prioritization of maternal and newborn health policies and their implementation in the eastern conflict affected areas of the Democratic Republic of Congo: a political economy analysis

**DOI:** 10.1186/s12961-024-01138-2

**Published:** 2024-04-30

**Authors:** Rosine Nshobole Bigirinama, Mamothena Carol Mothupi, Pacifique Lyabayungu Mwene-Batu, Naoko Kozuki, Christian Zalinga Chiribagula, Christine Murhim’alika Chimanuka, Gaylord Amani Ngaboyeka, Ghislain Balaluka Bisimwa

**Affiliations:** 1 Ecole Régionale de Santé Publique, Université Catholique de Bukavu, Avenue Michombero No. 02, Bukavu, Democratic Republic of Congo; 2 School of Medicine, Université Catholique de Bukavu, Bukavu, Democratic Republic of Congo; 3 Ecole de Santé Publique, University of Lubumbashi, Lubumbashi, Democratic Republic of Congo; 4 Airbel Impact Lab, International Rescue Committee, Nairobi, Kenya; 5 School of Medicine, Université de Kaziba, Bukavu, Democratic Republic of Congo; 6 Airbel Impact Lab, International Rescue Committee, Washington, DC United States of America; 7 Centre de Recherche en Sciences Naturelles, Lwiro, Democratic Republic of Congo; 8 Centre de Recherche Politiques, Systèmes de Santé, Santé Internationale (CR3), Ecole de Santé Publique, Université Libre de Bruxelles, Bruxelles, Belgium

**Keywords:** Maternal newborn health, Conflict, Health policies, Kivu, Democratic Republic of Congo

## Abstract

**Background:**

Maternal and neonatal mortality remains a major concern in the Democratic Republic of Congo (DRC), and the country’s protracted crisis context exacerbates the problem. This political economy analysis examines the maternal and newborn health (MNH) prioritization in the DRC, focussing specifically on the conflict-affected regions of North and South Kivu. The aim is to understand the factors that facilitate or hinder the prioritization of MNH policy development and implementation by the Congolese government and other key actors at national level and in the provinces of North and South Kivu.

**Methods:**

Using a health policy triangle framework, data collection consisted of in-depth interviews with key actors at different levels of the health system, combined with a desk review. Qualitative data were analysed using inductive and then deductive approaches, exploring the content, process, actor dynamics, contextual factors and gender-related factors influencing MNH policy development and implementation.

**Results:**

The study highlighted the challenges of prioritizing policies in the face of competing health and security emergencies, limited resources and governance issues. The universal health coverage policy seems to offer hope for improving access to MNH services. Results also revealed the importance of international partnerships and global financial mechanisms in the development of MNH strategies. They reveal huge gender disparities in the MNH sector at all levels, and the need to consider cultural factors that can positively or negatively impact the success of MNH policies in crisis zones.

**Conclusions:**

MNH is a high priority in DRC, yet implementation faces hurdles due to financial constraints, political influences, conflicts and gender disparities. Addressing these challenges requires tailored community-based strategies, political engagement, support for health personnel and empowerment of women in crisis areas for better MNH outcomes.

## Background

The high and disproportionate burden of maternal and neonatal mortality is a recognized problem globally, and particularly in low- and middle-income countries (LMIC) [1]. In 2021, the average maternal mortality ratio was 317 maternal deaths per 100 000 live births in Sub-Saharan Africa, which was almost 19 times the rate for high-income countries [2].The Democratic Republic of Congo (DRC) ranks fourth out of eight countries, reporting more than 50% of all maternal deaths worldwide, behind India, Nigeria and Pakistan [3, 4]. The country, specifically the eastern provinces of North and South Kivu, has been severely affected by more than three decades of armed conflicts; the negative effects of these conflicts on women and children’s health and wellbeing are profound [5, 6]. Insufficient healthcare funding and weak state authority, especially in conflict zones, worsen the health risks faced by these vulnerable populations [6–8]. The majority of maternal, newborn, and child health indicators remain poor in DRC. Indeed, although estimates for the period 2000–2017 showed an overall reduction of 38% in maternal mortality, this remains at 415 deaths per 100 000 live births in 2021. Neonatal mortality has been estimated at 22 deaths per 1000 live births in 2021 [9].

Interventions to prevent many of the maternal and newborn deaths already exist in the policies of many African countries, including the DRC [10, 11]. The reason for the deaths recorded would therefore not lie solely in the absence of policies, but perhaps also in the failure to implement these interventions or to adapt the content of global strategies and policies to the specific context [3]. Factors that hinder implementation may include a lack of political will, inadequate funding, insufficient capacity among health workers, power dynamics and poor governance and funding. In addition, regions with protracted conflicts, such as North and South Kivu in Eastern DRC, may present specific factors linked to the security crisis component. A better understanding of the relationships between these factors and decision-making in the MNH sector would enable policy development and implementation to be guided more effectively for the DRC context.

We have conducted a political economy analysis (PEA) [12] of policy development and implementation of MNH programs over the past two decades in the DRC. This PEA aims to understand the factors that facilitate or hinder the development of MNH policies, the prioritization of MNH, the decision-making processes and actor dynamics that determine the allocation of MNH resources and the implementation of MNH services by the Congolese government and other key actors at national and provincial levels.

## Methods

### Study design

We conducted a descriptive case study [13] that focussed on the PEA of MNH policies and their implementation in the DRC. We used a health policy triangle framework, which has been applied to PEAs in LMICs [12, 14]. The study period ran from October 2022 to August 2023.

### Study site

The study took place in the DRC, in the two eastern provinces of North and South Kivu, and in the capital, Kinshasa, where the national institutions are based. North and South Kivu provinces are among the country’s most severely affected by armed conflict, massive population displacement and complex socio-economic challenges [5, 8, 15–17]. In addition to the conflicts, these provinces are regularly hit by recurrent epidemics of infectious diseases and natural disasters, all of which contribute to the precarious health situation of the populations [18–22].

### Conceptual framework

**Fig. 1:**
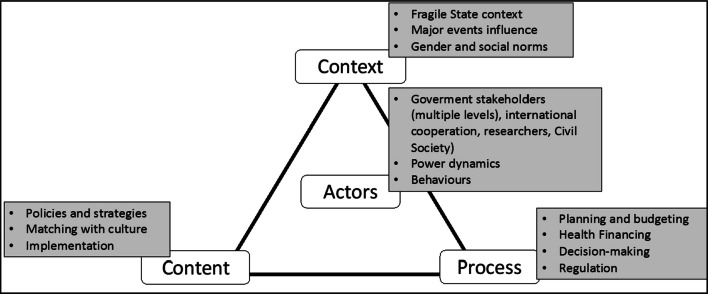
Health policy triangle framework adapted from Walt and Gilson (1994) [23]

We examined decision-making processes, actor dynamics and the underlying factors that lead to inadequate policy design, implementation and resource allocation – specifically for MNH within the eastern DRC context of conflict and fragility. Drawing on Walt and Gilson’s health policy triangle framework [23], the question was addressed by exploring the following themes (Fig. 1) through local, provincial and national levels and interactions across the three levels:

- *Policy-making process:* We examined planning, budgeting, health financing and decision-making processes influencing MNH policy implementation in crisis-affected areas of North and South Kivu.
- *Understanding policy content*: We assessed alignment of current DRC MNH policies with local values, health system and other factors affecting their execution in North and South Kivu.
- *Stakeholders and organizations*: We explored governmental and non-governmental actors impacting MNH prioritization and implementation across the DRC health system, analysing power dynamics and influencing factors.
- *Context and events*: We investigated how acute shocks, prolonged crises and local social norms affect MNH prioritization and execution in conflict-affected provinces.
- *Gender and social dynamics*: We integrated this theme into supporting question to understand how gender norms impact MNH prioritization.

### Case definition

The health system in the DRC functions across three levels: the Ministry of Health (MoH) at the central normative level, 26 coordinative Provincial Health Divisions in the 26 provinces and 516 operational Health Zones (HZ, or health districts) at the local level. Our study investigated the decision-making and implementation dynamics of MNH policies within conflict-affected regions, notably the North and South Kivu provinces. Our analysis covered the decision-making process at the central level, which impacts planning across the entire health system.

### Data collection

We explored the DRC and its eastern conflict-affected-area case through a desk review, as well as interviews with key stakeholders in the MNH space.

#### Desk review

We conducted a desk review of relevant academic, policy and technical reports from the health system and other relevant sectors (Ministry of Planning, Ministry of Budget and other government institutions), as well as international and national organizations playing a role in policy development and implementation. The PubMed MEDLINE and Google Scholar databases were used as the main source for relevant peer-reviewed academic articles. Article selection focussed on relevance to the research topic using the following keywords: Democratic Republic of Congo, maternal and newborn health, maternal death, health policies, Kivu, prioritization, conflict, low-income country. The desk review covered documents published between 2006 and 2021, except for key DRC Health System documents from earlier periods. We consulted a total of 36 peer-reviewed articles and 19 documents, which provided the primary basis for our analysis.

#### Key informant interviews

We conducted interviews with 24 stakeholders including political decision-makers. Interviewees were selected through a combination of purposive and convenient sampling. They included stakeholders from: the MoH, the Ministry of Planning, the Universal Health Coverage Fund and the Ministry of Budget [4], bilateral and multilateral organizations [4], technical staff in the MoH [6], researchers from the DRC with extensive international research experience [2], North and South Kivu civil society representatives [4], and healthcare providers working in the North and South-Kivu HZs [4]. A preliminary stakeholder mapping and analysis guided the selection of participants. The availability of participants also influenced the selection and the duration of data collection. While we aimed for gender balance in the selection of our interviewees, we only managed to interview 5 women out of the 24 interviewees. In this study, transcript validation with interviewees was not conducted.

We used semi-structured interview guides developed with guidance from the desk review and the conceptual framework. Data collection took place between October 2022 and August 2023. The interviews were conducted face-to-face or online via Zoom Video®.The interviews were conducted in French and recorded. They lasted an average of 43.4 min. Four members of the research team conducted the interviews (R.B., P.M., C.Z.C. and G.N.).

### Analysis

To analyse the transcriptions, we followed the reflexive thematic analysis approach [24]. We first became familiarized with the data by reading and re-reading the transcripts and discussing the potential themes that were prevalent in the data with the team. In the second step, we created codes related to the common responses from the transcripts. After a structural coding from the interview guides, we developed descriptive codes within each group of responses. Through line-by-line coding upon further reading of the responses, we generated subjective codes. In the third step, pattern codes were created to categorize initial codes and generate themes. The team then consultatively reviewed themes, discussing their meaning and taking into consideration the richness of data associated with them. In step five, the team defined the themes, interpreting the main findings and selecting extracts from the data that demonstrated the main findings. Step six consisted of reporting: findings were written up according to the study frameworks, with co-authors collaboratively deciding on main arguments to be derived from the findings in relation to key drivers of MNH prioritization in DRC.

The analysis was carried out iteratively and collaboratively. The structural, descriptive and line-by-line coding were prepared by R.B. assisted by C.Z.C. After this point, they were joined by five other researchers (PM, CMC, GN, MM and GB) to collaboratively conduct the pattern coding and deductive incorporation of themes into the conceptual framework. We conducted a second literature search to assess the applicability of the deviant codes and findings that did not fit into the main categories. We were then able to understand and interpret how these deviant codes and findings can be integrated into the analysis.

### Ethical considerations

This research was approved by the ethics committee of the Université Catholique de Bukavu under the reference UCB/CIES/NC/021/2022. All interviewees voluntarily gave their informed consent prior to the interview and audio recording.

## Results

### The process of developing and implementing MNH policies

#### Strategic policy planning at national level

Respondents stated that policy development is driven primarily by identified health needs, aligning priorities with available resources and government directives. Crucial data from surveys such as the national Demographic and Health Survey and the Multiple Indicators Cluster’s Survey helps establish these needs, but our review found that the latest surveys were in 2018 and 2014 respectively, while the last national census was in 1984. According to respondents, limited financial resources hinder the government’s independent studies, emphasizing the role of scientific research, often conducted via international collaboration or by local researchers, in guiding decisions.

The MoH plays an essential role in prioritizing MNH since its advocacy can shift the balance in favour of prioritizing funding for the sector’s policies. According to respondents, the MoH currently focusses its advocacy on MNH policies. Despite this consideration, health financing for MNH in DRC faces major challenges. The country has not yet reached the 15% threshold of the budget it committed to allocate to health [25]. Challenges contributing to poor allocation include insufficient government revenues, largely due to corruption, delays in revenue collection and the big size of the informal economy.

*A lot of people are criticizing the (national) budget. This budget is not realistic. (The allocated amounts) don’t really reflect the budget and it doesn’t really reflect the resources. The resources are minimized and so are the needs... And when we contribute, what do those who receive the money do with it? You’ll see someone who is this [an ordinary public officer] but he lives a high-class lifestyle and has villas. He’s a big boss. And then there’s the political involvement, the exonerations that are made, the influence peddling, the weakness of the justice system... all these elements contribute to the fact that we can have a budget that is not consistent. (Respondent 3/Central level)*

*Yes, the budget is insufficient, that’s a fact. The budget depends on revenue, and revenue often doesn’t keep up for several reasons... there’s a regulatory malfunction in the various sectors, and that means that revenue doesn’t keep up. That’s on the one hand, and on the other, revenues are not keeping pace because of bad practices. There are people who think only of their bellies. There’s embezzlement, people don’t pay their taxes. All this means that the State doesn’t have the means to implement its policies. Thirdly, the informal economy accounts for over 80% of employment in the DRC. These people are not indigents. They collect money but don’t pay taxes. (Respondent 2/Central level)*

Setting priorities in the MNH area is also complex because of the diversity of issues in the health sector, all of which are considered equally important and affected by the government’s insufficient funding of the health sector. Donors and households therefore play a significant role in financing healthcare (37% and 43%, respectively) [25]. In the end, prioritization ends up falling in the hands of donors, depending on the resources available from existing partnerships.

*It’s still difficult because we still have the impression that everything is a priority. That’s why I said to you: sometimes you have funds, and you ask yourself, where do we start, what’s going well, what’s not going well? But of course, there is well-targeted support, there are partners involved in planning for safer motherhood, for violence, etc. (Respondent 7/Central level)*

Health policies in the DRC are thus influenced by international partners, with diverse funding sources and new partners redirecting resources to MNH. The Kinshasa Agenda Initiative in 2010 and other initiatives such as the donor Groupe Inter-Bailleurs Santé (GIBS) coordinating structure [26, 27] have facilitated international aid coordination in the health sector. The Global Financing Facility (GFF) [28] played a key role in reshaping the National Health Development Plan into a MNH-focused plan in 2018.

#### Planning and implementation of MNH strategies in the provinces

The implementation of MNH policies involves collaboration between the central and operational levels. Local political, administrative, cultural and religious authorities all play key roles in supporting implementation of MNH initiatives. Respondents generally appreciated the quality of communication across the three different levels, but few concerns have been raised by HZ actors who desire more consistent feedback from higher levels. Adapting central guidelines to suit the unique requirements of local communities also poses a significant challenge for the provincial and HZ actors in North and South Kivu. However, it happens sometimes that MNH activities funded by external partners are directly locally planned, which gives opportunity to HZs to formulate strategies tailored to their specific context and based on local evidence.

### Policies content and the UHC in the DRC

#### MNH policies in DRC over the last two decades

**Fig. 2:**
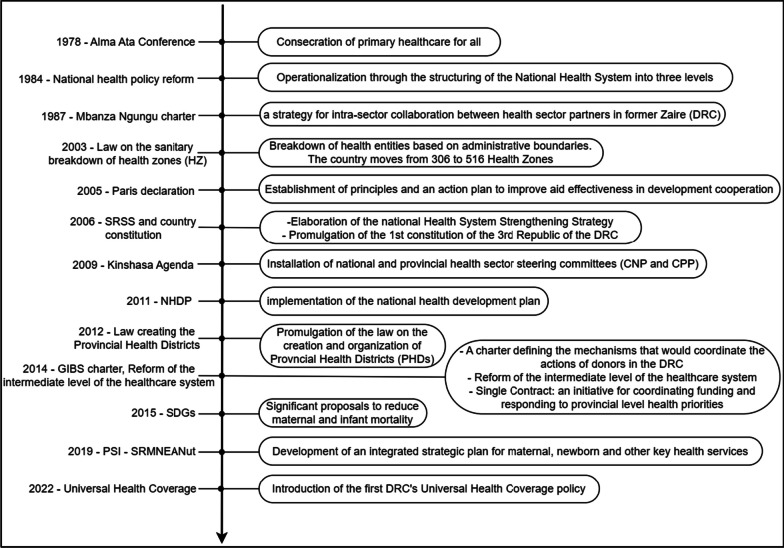
Key evolution of MNH policies in DRC

According to respondents, MNH is sufficiently a priority at national, provincial and local levels. Policies governing MNH in the DRC (Fig. 2) are organized through the Integrated Strategic Plan for Reproductive, Maternal, Newborn, Child and Adolescent Health and Nutrition (PSI SRMNEA-Nut), a plan intended to be updated every 5 years [29]. Reducing maternal and neonatal mortality was a recurring theme in discussions with our respondents, underlining the importance of MNH currently in the Congolese health system.

*So, here in Kinshasa, it’s such a priority... It’s already a priority at national level… all the strategic documents today focus first and foremost on maternal and child health. Since the pace is already set at national level, even more so when you go down to provincial level, to zone level ... in my observations I can say that the providers, yes, they also make it a priority, because the providers implement a policy (that was elaborated) at national level. (Respondent 23/Health partner)*

*It’s fair to say that maternal and newborn health have a special place in custom, because it’s not easy for a woman who can’t have a child to be accepted in the community. And even for the structure when there is a maternal death, there are enormous conflicts. Even in conversation between women, you can feel that motherhood has a special place. (Respondent 19/Health zone).*

#### The universal health coverage (UHC) policy in DRC

The prioritization of MNH related laws and policies can sometimes be shaped by the influence of the head of state. This was the case with the UHC policy, which the current president made a priority by placing it on his electoral portfolio. The related law No. 18/035 was enacted in December 2018, and implementation begun in 2019. Despite the presence of the law, however, it was only in March 2023 that the president signed an ordinance establishing a health insurance scheme to ensure financial protection under UHC. A free childbirth program has been launched in one province by the head of state on 5 September 2023, with plans for nationwide expansion [30]. The newly formed health insurance system is yet to expand to the rest of the country, so much of the country is still without formal health insurance system. There have been a few attempts to get communities to join local mutual health insurance schemes, but their impact on the country as a whole remains elusive [31]. Patients pay directly out of their own pockets for care in health facilities, either on a fee-for-service basis or by paying a fixed fee, which may sometimes be partially subsidized by international partners from bi- and multi-lateral cooperation [31, 32]. The challenges faced by the UHC policy include funding, human resource management and the need to ensure effective implementation of free MNH care.

Although MNH service provision has been made free, respondents state that public health structures needed to provide quality healthcare are still below standard. This provides an opportunity for collaboration between the public health sector and the private sector which has been integrated to the national health system. This private sector has proved to be a strong support for healthcare provision in DRC [33].

*... when we toured all the health zones (in Kinshasa) with the Minister of Health, our visits showed that there was a problem with public health facilities... to implement the Head of State’s policy, we need to improve the quality of care in public facilities. That’s the major challenge, because we’ve seen that denominational health facilities, especially Catholic ones, and private facilities offer a quality package. (Respondent 6/Central level)*

### Role of stakeholders in the development and implementation of MNH policies

#### Power dynamics among actors in the MNH sphere

Stakeholders including government entities, national and international partners, and civil society play different roles in the DRC’s MNH environment. The national MoH holds considerable decision-making power. Public health technical staff collaborate closely with international partners in policy development and implementation and receive validation from the national MoH. The provincial health minister mirrors his national counterpart and reports to him. Both ministers are appointed by the government. Changes in government are frequent in DRC, and always lead to new ministers of health. Respondents reported that these changes, observed thrice in the past 5 years, often cause disruptions in stability of ministries and staff motivation within the sector, as they often come with technical staff reshuffles.

International bodies such as the WHO are involved in planning and implementing MNH policies at all levels. They support the development of health recommendations and guidelines at the central level. They also provide, as mentioned above, funding support to improve the accessibility and quality of care in public establishments in the provinces. Some respondents see them as holding the greatest decision-making power, given the country’s dependence on external aid.

*Quite simply, the health sector is dependent on international aid, which I think also affects the country’s leadership. Leadership also affects governance: you see, a partner who gives you more, you’re going to regulate it, but with reservations. Otherwise, when you’re distracted, it’s the partner who starts laying down the law, because he’s contributing, he’s bringing in a big envelope. (Respondent 8/Central level)*

*“The donors obviously have the money, so we have to follow those who have the money. And I think that even the government follows”. (Respondent 12/Civil society).*

The government has established collaborations with various private sector players with the aim of extending the coverage of maternal and newborn healthcare. Among them, confessional private organizations have played a major role in the provision of MNH services for many years [33]. In general, these entities operate under state approval and are part of the formal healthcare system. Nevertheless, certain private entities operate without licenses and integration, offering subpar healthcare, notably in the MNH domain. While regulatory standards exist to oversee such private actors, implementing effective monitoring faces challenges, primarily due to resource constraints and pervasive influence peddling practices. This situation often places these unregulated private entities beyond the reach of local regulatory authorities.

*Well, apart from the staff, the other challenge, and this is perhaps the greatest challenge, is that the private structures arrive in the zone without going through the HZ central office... They arrive with documents. We don’t know whether they are real documents or not. And when they arrive, they bypass the central office. They go straight in. When we want to go after them, we don’t have the police or the army, so they stay there like a so-called health structure, but they don’t respect anything. (Respondent 21/Health zone)*

#### Culture and actor motivations, interests and ideas

Several cultural influences can positively or negatively impact adherence of communities to MNH policies. Traditional beliefs, social and religious norms and language barriers have significantly influenced the success of MNH interventions. For example, some communities favour traditional practices over modern medicine despite the potential risks.

*You also know in the same areas of [names redacted, Kivu territories], it is thought that when a child is born, to make it strong it should be bathed in cold water. And in the same health zones they think you should put dung on the umbilical cord. (Respondent 16/Provincial level)*

*“... no woman here can go to antenatal cares at 16 weeks because, to announce a pregnancy in our environment, it has to be (physically) visible to everyone." (Respondent 19/Health zone)*

Prayer rooms and traditional healers are also a challenge for HZ, as they have a proven ability to dissuade women from seeking medical care. In particular, in rural areas of Kivu, a prevalent belief called *sanga* prevents pregnant women from seeking the assistance of qualified personnel for deliveries.

*When you go to [health zone’s name redacted]. For example, you’re told that when a woman goes into labour and her husband has cheated on her (during her pregnancy), if she doesn’t take the sanga, she won’t give birth … some women refuse the reference outright. She says, ‘I know it’s sanga and I’ve already taken the traditional medicine, I’m going to give birth’. And even when a referral is deemed necessary, they prefer to go to another house of traditional practitioners and charlatans where they will put her on hold and wait until she gives birth. (Respondent 16/Provincial level)*

*We can tell a woman that her pregnancy is at risk and that she needs to go to a facility to be monitored by a doctor, but the prayer rooms and traditional healers will tell her that everything will be fine. They’ll pray for her and give her indigenous products, but in the end it all stalls. (Respondent 19/Health zone)*

However, some respondents noted the useful role that traditional practitioners could play as collaborators in the promotion of MNH, provided they were properly supervised.

*I also remember in the territory of [name redacted] we discussed with women who give birth at home and who continue to provide care at home, we discussed with the traditional authorities and after the advocacy it is the traditional authorities who force these women so that even if they have given birth at home, they still go to the health centre. (Respondent 16/Provincial level)*

Alongside these challenges, MNH appears to hold limited significance among male members of the local community, given their inadequate involvement as partners in women’s health.

*Usually speaking, maternal and newborn health is not really a priority for the local community here. Why am I saying this? You see, for example, there’s antenatal care for fathers, which means that men must accompany their wives to antenatal care. The percentage is extremely low. To say, ‘It’s your pregnancy, go and do your tests, it’s your pregnancy’. You understand that it is not really understood in the community that it’s really a family problem, that it’s a problem of both partners. It becomes a woman’s problem with her child... (Respondent 20/Health zone)*

### Influence of context and major humanitarian events on MNH

**Fig. 3:**
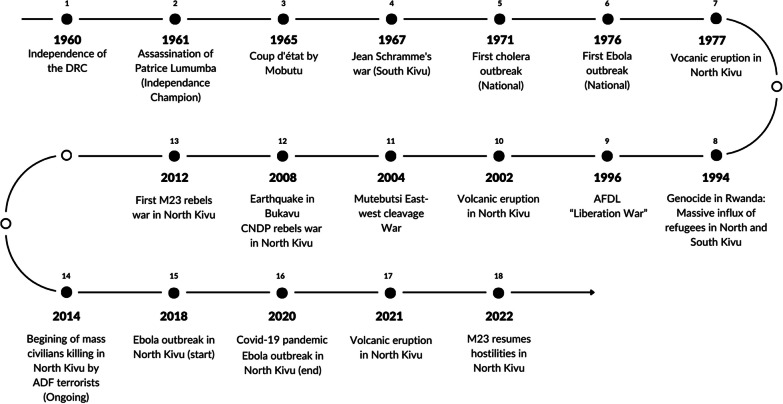
Major health, environmental and security events affecting eastern DRC since independence

The figure below (Fig. 3) illustrates the major health, environmental and security shocks that the health system in the DRC, and in the two provinces of Kivu in particular, has had to deal with over time, and which have had a profound effect on its organization.

The DRC’s expansive size poses challenges since many regions are almost inaccessible due to inadequate transport and communication infrastructure, impacting overall country management [5]. The vital support to MNH from provincial health coordination and external partners can also be limited in some areas due to geographical or security obstacles.

*The [redacted] health zone has no partner for reproductive health apart from the Provincial Health Division... we are told that it is accessibility that is complicated because the [redacted] health zone covers an area of 6500 km*^*2*^* and access is not easy. Sometimes partners compare the cost of logistics and say to themselves that the same activity they can carry out in my zone would have a greater impact if it were carried out in another zone. As a result, many partners refuse to come to my area because of the state of the roads. (Respondent 19/Health zone)*

*We have geographical barriers; health zones such as [redacted], [redacted], [redacted], it’s very difficult to access them... there are zones where we do even a year without getting there [for a supervisory visit] and when we arrive, we’re only going to focus on the general referral hospital in the zone and the health zone but we can’t go to the facilities. (Respondent 16/Provincial level)*

Provincial health providers in North and South Kivu areas partly occupied by rebel militias are obliged to develop mechanisms for peaceful cohabitation with them to maintain the provision of MNH care.

To enhance responsiveness during medical emergencies, aid organizations such as the World Bank occasionally supply ambulances and motorcycles. However, these emergency vehicles do not reach all HZs in North and South Kivu [34, 35]. Infrastructure challenges, as highlighted by our respondents, primarily hinder the distribution of these vehicles in the Kivu:

*‘If there are no roads, the ambulances won’t be able to get there. The proof is that we have the ambulance from [redact] here, it failed to reach to [redacted, a North-Kivu Health Zone]. It's been here for two years’. (Respondent 15/Provincial level)*

According to our respondents, fragility caused by protracted conflict alongside acute crisis present challenges, particularly in terms of logistics and resource mobilization, as well as in regulating the health sector.

In the DRC, acute crises such as epidemics, wars or natural disasters profoundly affect decision-making and funding for MNH services, further straining already limited resources. Some regions lack the necessary logistics and infrastructure to manage emergencies, and there are no established guidelines for such situations.

*But it must be said clearly that, once again, in the [redacted] health zone, this (emergency service) approach has not yet been implemented. MNH services are provided there, but as I said, perhaps the quality isn’t guaranteed... in crisis, they don't know how to provide this continuity because they don’t have the capacity... there’s nothing pre-positioned, either in terms of kits or the management itself, in a crisis, they don’t have the capacity to provide the minimum emergency services. (Respondent 16/Provincial level)*

During major humanitarian events, the healthcare system is temporarily dysfunctional. Moments of health system dysfunctionality during major humanitarian events serve as an opportunity for some private, unlicensed, and non-integrated providers to bypass the public health system in order to create illegal parallel structures which may even last beyond the momentary humanitarian disruption.

*...during the crisis the health system in the affected area is overwhelmed, so we’re adopting a humanitarian approach. But although there is a humanitarian approach, we must make sure that the health system does not deteriorate. Because that’s often what happens, (individuals) start to bypass the system, they start to create parallel structures. It shouldn’t be like that. (Respondent 8/Central level)*

However, it appeared that crises can also give rise to positive impulses to mobilize players and resources. They can also provide opportunities to implement strategies and initiatives to promote MNH.

Very often, an emotional trigger can make an issue become a global priority due to extensive media coverage: epidemic, armed clashes in a civilian area, volcanic eruption or major earthquake. Such mobilization often proves transient, tied to the crisis itself, and thus focussed on immediate relief rather than sustained development. During the 2018 Ebola outbreak in Equateur province, north-west of the country, the MoH, funded by the World Bank, implemented a policy providing free healthcare to the affected and nearby populations. A study revealed the policy’s positive impact on the use of MNH services, but this effect waned once the specific epidemic ended [36]. Some respondents suggested that, to sustain the positive initiatives generated by crises, the MoH should take advantage of the influx of funds during crises to rehabilitate health infrastructures and encourage community ownership. Other respondents noted that armed conflicts, requiring a war effort, can be competing priorities with health priorities when resources are limited.

*‘As long as we don’t have security, we’re going to prioritize buying weapons and sending troops to the front and spend less on investing in social sectors like health and others’. (Respondent 5/Central level)*

Despite the challenges posed by epidemics, wars and natural disasters, institutions and organizations working in the field of MNH in North and South Kivu strive to maintain continuity of services by raising awareness in local communities, repairing damage caused by disasters and seeking ways to access patients whenever possible. Communication and diplomacy remain a key strategy for dealing with these crises.

*...what we do first is to maintain contact with all the structures. For example, when there’s insecurity, we maintain contact with all the authorities involved; that means the leaders of the armed groups... For example, if there’s a woman we need to rescue and we can get there, in any case we ask (to the rebels) for authorization to go and take or treat these women where they are. (Respondent 20/Health zone)*

### Gender and equity as MNH factors

The importance of the participation of women and minorities (people with disabilities, displaced persons and ethnic minorities) in MNH planning and services was recognized by respondents. However, there are still challenges in the form of gender norms and cultural stereotypes that maintain the glass ceiling on achieving the normative ideal of 30% female representation in public institutions, as guaranteed by Article 14 of the DRC Constitution. [37]. Respondents also mentioned persistent tendency in local cultures to prioritize the education of male children over female children. The result is a shortage of competent female staff who can compete with their male counterparts. This, along with fear of working in unsecure areas [38], has an impact on the composition of the staff providing MNH services: there are few female care providers.

Similarly, in decision-making positions, our respondents noted that women are poorly represented.

*If we look at maternal health, for example, I noticed that the heads of the health divisions in South Kivu and North Kivu are men. They’re the ones who are talking about the problem of maternal and newborn health, so these are positions that I think would be better suited to women... they’ve held these positions for several years now, and a woman has never been appointed to them. And if, at provincial level, the authority in charge of maternal health is a man, I think that the structure down there will also discriminate against women and certain gender-specific needs of women will not be met in the same way as if a woman were in that position. And now if we can take that up to national level, it would be the same thing, which means that policies won’t change in the same way if we just take the men’s view, how men see the problem, rather than how men and women approach the problem in order to find changes. (Respondent 12/Civil society)*

## Discussion

The aim of our study was to explore the factors involved in the prioritization and implementation of MNH policies in DRC. We analysed the interactions between these factors at different levels, from national to local, through the Walt and Gilson’s health policy triangle framework [23].

### Overall challenges

Our study uncovered multiple challenges that hinder the effective prioritization and implementation of MNH policies. These challenges span from competing health emergencies and limited resources to political influence. Additionally, socio-cultural beliefs and the impact of major crises further compound the complexities of delivering MNH services in eastern DRC’s crisis context. Despite efforts to adapt policies to local contexts and promote gender equality in decision-making, significant disparities persist. The evolving political landscape and ongoing armed conflicts also present dynamic challenges. In the following discussion, we delve deeper into these challenges and explore potential pathways to address them.

### Setting priorities

According to our findings, prioritizing MNH policies in DRC is challenging due to diverse competing health emergencies, with limited public funding and poor financial management systems. Prioritization relies more on resource availability and external partners agendas. The sector is therefore driven by short-term action plans over comprehensive, integrated strategies. This echoes the challenge faced by many low-income countries, most of which depend on foreign aid [39–41]. Other researchers have also observed the presence of actors with competing priorities, limited and often poorly distributed financial and human resources, and systemic corruption among the factors influencing the prioritization of MNH policies in similar contexts [42, 43]. Prioritization under these conditions will only be effective when different independent streams coincide at a point of common interest, through better coordination [44, 45]. While current initiatives aim to harmonize efforts among partners in the DRC, there remain lingering doubts and insufficient evidence about their effectiveness in achieving comprehensive coordination [27]. Further evaluation is required to assess the sustained impact of these coordination initiatives on improving the prioritization of MNH policies.

### Adapting policies to local contexts

According to our findings, central guidelines are established on the basis of evidence from local demographic surveys to ensure their alignment with local community needs. Additionally, some activities are locally planned and executed with external partner funding, enabling HZ to create strategies directly on the basis of local evidence. Several studies in similar contexts have described how community-based approaches to improving MNH were better received by local populations, thus increasing their chances of success. Indeed, in addition to meeting community expectations, community-based health planning and services promote trust between community members and decision-makers [46–48]. Existing research shows that a lack of trust within the community can significantly undermine the effectiveness of health promotion strategies [49]. In the context of the DRC, challenges such as the vastness of the country, its remarkable cultural diversity and the multitude of distinct local contexts, and particularly the protracted crisis context in the country’s eastern provinces, are further amplified by resource constraints [50, 51]. These challenges need to be considered during the planning process and necessitates flexibility in MNH investments.

### Political influence of individuals

Our findings regarding the prioritization of UHC in the DRC are consistent with the current broader global discourse on UHC as the dominant paradigm of health policy as literature report [52, 53]. The emergence of UHC as a hegemonic policy, as highlighted by Smithers and Waitzkin [54], shows the significant influence exerted by international financial institutions, in shaping global and country health agendas. This resonates with our findings that uncovered the influence of international partners in DRC when setting priorities.

Our results show that the UHC policy aiming to ensure equity in MNH service access is being politically prioritized by the head of state. The literature describes how political champions are essential to health policy prioritization and implementation of policies [55, 56]. However, the fact that a policy is promoted by a political champion can be the cause of the policy’s failure, regardless of its potential effectiveness. This was the case in Uganda, where a health program specially coordinated by the presidency was contested because it was perceived as political interference [57]. Another detrimental mechanism for the intervention of political champions in health policies could result from the phenomenon of path dependence, described when major success of large-scale initiatives strengthen the credentials of previous choices and prevent decision-makers from exploring new alternatives in future scenarios [58, 59]. Accompanying system-oriented measures should be attached to policies backed by a political champion to guarantee their sustainability even beyond the time of their initial bearer.

Furthermore, the literature suggests that the concept of UHC is often poorly or not at all defined, leading to wide variations in its interpretation, and consequently, in its implementation [54, 60, 61]. This lack of clarity seems evident in our examination of the policy-making process in DRC, where low state funding for health may lead one to question the sustainability of this UCH policy. Ultimately, UHC could therefore become a hollow brake on other effective non-insurance-based approaches, if policy-makers do not insist on the budgetary measures that will have to accompany it.

### Staff turnover

Our study finds that the frequent government changes often result in new appointments of ministers, which can disrupt the sector’s stability and the motivation of staff. The loss of experienced professionals in the field because of changes in government can erode the continuity of programs and specialized knowledge and skills needed to meet the challenges of MNH [62]. As a result, it can hinder the progress and effectiveness of MNH initiatives. It is described how many health initiatives in Zimbabwe failed due to the political instability that hit the country in the 1990s as civil wars raged. The failure was linked to the absence of mechanisms for continuing these initiatives. Projects were abandoned when their promoters left due to political unrest [63]. Strategies to preserve expertise and ensure continuity of efforts to improve MNH performance are needed at all levels in DRC.

### Health operators’ influence beyond state authority

Our results showed that some private providers operate in the shadows and provide low-quality care at lower prices. This phenomenon is more prevalent in crisis areas where access is limited due to geographical and/or security difficulties, and where the state fails to enforce its power. These unlicensed providers mainly target women from the most vulnerable socio-economic categories and bypass local regulatory authorities through influence peddling. As the choice of place of delivery is often linked to women’s degree of autonomy [64, 65], literature reports how interventions aimed at empowering women, particularly at the social and educational levels, have had a direct positive impact on MNH [66, 67]. In the context of LICs, empowerment of women is crucial, yet decisions on healthcare might encompass factors beyond cost, including accessibility and perceived quality, influencing their preference for some facilities, regardless of the objectivized quality of services, as authors have observed in similar areas [68]. The government should prioritize providing financial protection or accessible, pro-poor healthcare systems in these areas where the state has little presence [69, 70] while continuing to try to implement legislative mechanisms to discourage the unlicensed sector [71].

### Socio-cultural concerns

We have found that client’s resistance to the implementation of MNH policies may result from cultural beliefs, social norms and traditional practices, among other factors. Meanwhile, traditional practitioners could play a useful role in promoting MNH if properly integrated, according to our findings. The negative influence of traditional healers and ancestral culture on MNH has been demonstrated in various contexts in low-income countries [72]. Health sector actors can address this issue by working on four key dimensions: cultural competence, community engagement, education and awareness, and supervision and collaboration. Cultural competence is essential for understanding socio-cultural barriers and respecting local practices, while community involvement promotes community awareness and the fight against aberrant beliefs [73, 74]. Supervision of traditional practitioners, as well as collaboration between modern and traditional providers, have proved their worth in other countries [75]. It should be remembered, however, that the described practice of influence peddling remains a factor that can potentially undermine the health sector’s efforts to regulate traditional practitioners, as our results have shown.

### MNH care delivery in conflict zones

We found that providers in areas partly occupied by rebel militias have to establish mechanisms for peaceful cohabitation to ensure the continued provision of MNH care provision. Few female MNH providers are willing to work in these areas because of the insecurity. In addition to insecurity by itself, studies have identified health workforce issues such as lack of skilled health staff, epidemics such as coronavirus disease 2019 (COVID-19) and Ebola, and financial constraints as barriers to MNH care delivery in conflict zones. They have also emphasized the pressing need for more research to evaluate the delivery of MNH interventions in these challenging settings [76–79]. Violence against health workers in conflict zones is a reality described by several authors [80]. When conflicts in these areas drag on, health professionals develop a variety of resilience mechanisms. These range from strategies aimed at reducing direct risks to negotiation efforts aimed at establishing peaceful coexistence with the armed groups occupying the region [81]. However, while this demonstration of resilience is generally perceived positively, co-habiting with entrenched armed groups in a region exposes healthcare providers to a constant risk of being abducted and forced to provide services on behalf of these armed groups [82]. What’s more, there is a risk that the apparent stability resulting from the conciliation between healthcare providers and rebels could in the long run serve as an excuse for health officials to ignore the difficult situation of this front-line staff. By abandoning health personnel to their own devices in such contexts, the quality of healthcare, and in particular of MNH care provided to the population in the area, could suffer in the long run [83].

### The influence of major crises

Acute crises can paradoxically create opportunities for mobilizing resources and implementing MNH strategies, but crisis-related funding often prioritizes immediate relief over sustained development, as our results show. Some sources note that crisis and immediate post-crisis periods offer an opportunity to reduce gender prejudice and stereotypes and implement impacting MNH and general health promotion actions [36, 84, 85]. However, other authors point out that, in times of crisis, donors tend to focus narrowly on programs and interventions linked exclusively to women’s reproductive health, rather than on the broader maternal, newborn and reproductive health. [36, 86]. In all cases, these interventions generally last only as long as the crisis. Yet major crises offer a unique opportunity for the healthcare system, bringing together three key elements, as emphasized by some authors [87]. These elements include a significant influx of external funding dedicated to crisis management, the presence of experienced health workers on the ground ready to intervene and the overabundance of health graduates on the market. Therefore, what is generally considered a negative shock to the healthcare system could, in these circumstances, turn out to be a policy window [56] for sustainable system improvement, provided that sector decision-makers consider a mechanism for capitalizing on this opportunity. Further research could delve into exploring how healthcare systems in crisis settings can effectively capitalize on such policy windows to drive sustainable MNH improvements amidst challenging circumstances.

### Gender in the MNH decision-making space

We found a noticeable gender disparity in decision-making positions related to MNH in DRC. Women are poorly represented in these positions. Even with our selection of respondents, we were able to achieve barely 20% female representation despite our desire to highlight female voices. Studies in resource-limited settings have highlighted the lack of medical staff, particularly women, as a factor hampering the prioritization and provision of maternal health care in crisis zones [43, 88]. Women are often under-represented due to constraints related to professional hierarchy, gender-based cultural barriers and security constraints, particularly in remote and insecure areas as mentioned above [86, 89]. When women are under-represented in decision-making positions, this can result in policies and strategies that do not fully address women’s unique healthcare needs, particularly in MNH. The literature demonstrates the benefits of involving women in decision-making positions, notably linked to the fact that leaders tend to favour more strategies that directly address the needs of their own gender [90, 91]. More research is needed on gender main-streaming mechanisms in DRC and their potential impact on improving MNH, especially in the context of health system strengthening via UHC.

### Study implementation context

At the time of writing this study report (early December 2023), the North Kivu province is under a state of siege and active armed clashes since the end of 2022, and national presidential elections are planned for 20 December 2023. The evolving political landscape post-election may alter policy directions and healthcare priorities, potentially impacting the applicability of the study’s conclusions in the longer term. However, it is important to note that this represents an opportunity for future research rather than a strict limitation. As the world is in constant flux, research studies have inherent time constraints, and while this aspect influences the study’s scope, it also provides scope for ongoing exploration and analysis. Additionally, situational context markers, such as the active conflict in North Kivu and upcoming election timelines, must be acknowledged as they may affect the immediate relevance of the study’s findings.

### Other limitations

While our study aimed to provide an in-depth analysis of the MNH policy environment in the DRC, certain limitations should be acknowledged. Firstly, the study duration, conducted from October 2022 to August 2023, will not capture all ongoing policy changes or the full scope of implementation dynamics that could evolve post-data collection. Additionally, the persistent armed conflict in North Kivu during the study period added another layer of complexity. This currently still ongoing conflict may have potentially affected certain perspectives.

## Conclusions

Our study suggests that MNH is a high priority in DRC in terms of political will and development of relevant policies. However, the effective implementation of MNH policies is hampered by the multiplicity of issues, including insufficient public sector funding, sub-optimal financial management of state resources and the impermanence of external partner investments. Matching strategies to the needs of local communities, political influence at all levels, the issue of the resilience of first- and second-line services in areas of active conflict and the fight against gender disparities in decision-making are essential factors to be considered when setting priorities for MNH. Our analysis also offers insights that extend beyond the context of the DRC, shedding light on the broader challenges that impede the achievement of UHC in other low-income countries grappling with conflict and humanitarian crises. For better MNH results, it is necessary to develop flexible, community-based strategies, to engage judiciously with political influencers, to maintain the stability and decision-making autonomy of health personnel and to empower women in communities, particularly in crisis areas. Additionally, increasing domestic health financing is essential to strengthen health infrastructure and services, thereby enabling more effective implementation of MNH policies.

## Data Availability

Data generated and analysed during the current study are not publicly available due to confidentiality restrictions, but anonymized transcripts are available from the corresponding author upon reasonable request.

## Abbreviations

DRC: Democratic Republic of Congo
LMIC: Low- and middle-income countries
HZ: Health zone
MNH: Maternal and newborn health
MoH: Ministry of Health
PEA: Political economy analysis
UHC: Universal health coverage
